# Sex-specific differences in trimethylamine N-oxide (TMAO) concentrations before and after cardiac rehabilitation in acute myocardial infarction patients

**DOI:** 10.17179/excli2021-4366

**Published:** 2022-01-03

**Authors:** Andreas Baranyi, Andreas Meinitzer, Dirk von Lewinski, Hans-Bernd Rothenhäusler, Omid Amouzadeh-Ghadikolai, Hanns Harpf, Leonhard Harpf, Heimo Traninger, Ronald Hödl, Birgit M. Harb, Barbara Obermayer-Pietsch, Melanie Schweinzer, Celine K. Braun, Dietmar Enko

**Affiliations:** 1 Department of Psychiatry and Psychotherapeutic Medicine, Medical University of Graz, Graz, Austria; 2 Clinical Institute of Medical and Chemical Laboratory Diagnostics, Medical University of Graz, Graz, Austria; 3 Division of Cardiology, Department of Internal Medicine, Medical University of Graz, Graz, Austria; 4 Department of Psychiatry and Psychotherapy I, State Hospital Graz II, Graz, Austria; 5 ZARG Zentrum für ambulante Rehabilitation GmbH, Graz, Austria; 6 Ordination Hödl, Ordinationszentrum Privatklinik Graz Ragnitz, Berthold-Linder-Weg 15, 8047 Graz, Austria; 7 Pensionsversicherungsanstalt, SKA-RZ St. Radegund für Herz-Kreislauferkrankungen, St. Radegund, Austria; 8 Division of Endocrinology and Diabetology, Department of Internal Medicine, Endocrinology Lab Platform, Medical University of Graz, Graz, Austria

**Keywords:** Trimethylamine N-oxide (TMAO), acute myocardial infarction, sex differences

## Abstract

Trimethylamine N-oxide (TMAO) is a biomarker of cardiovascular risk and may enhance the progression of atherosclerosis. The aim of the study was to determine whether there are sex-specific differences in TMAO concentrations before and after cardiac rehabilitation in acute myocardial infarction (AMI) patients. A total of 56 participants [45/56 (80.4 %) males, 11/56 (19.6 %) females] were drawn from AMI inpatients hospitalized at the Division of Cardiology, Medical University of Graz, Austria. For the assessment of TMAO, serum samples were collected within the first day after hospital admission due to AMI and at the start and end of cardiac rehabilitation. Shortly after hospital admission due to AMI, females had significantly higher TMAO blood concentrations than males. These initially high TMAO levels remained almost unchanged in the female AMI patients until the start of cardiac rehabilitation and only reached the lower TMAO concentrations observed in the male patients after rehabilitation [female patients: TMAO (acute myocardial infarction) = 5.93 μmol/L (SE = 1.835); TMAO (start of rehabilitation) = 5.68 μmol/L (SE = 1.217); TMAO (end of rehabilitation) = 3.89 μmol/L (SE = 0.554); male patients: TMAO (acute myocardial infarction) = 3.02 μmol/L (SE = 0.255), TMAO (start of rehabilitation) = 3.91 μmol/L (SE = 0.346), TMAO (end of rehabilitation) = 4.04 μmol/L (SE = 0.363)]. After AMI, women might be at higher cardiovascular risk due to persistently higher levels of TMAO. High TMAO levels in women might decrease after cardiac rehabilitation due to cardiac rehabilitation-associated lifestyle modifications. These lifestyle modifications after AMI might also prevent increases in TMAO concentrations in men.

## Introduction

### Sex-specific differences in acute myocardial infarction (AMI)

Acute myocardial infarction (AMI) is the leading cause of death and disability in US women (Mehta et al., 2016[16]; Andersson et al., 2007[2]). In this acute cardiac emergency, the blood supply to the heart is suddenly interrupted by a blockage in the coronary arteries. As a consequence, cardiac muscle cells die due to the lack of oxygen and nutrients. Therefore, it is essential to recognize the symptoms of AMI as early as possible. However, AMI is more frequently misdiagnosed due to the greater variability in symptom phenotypes in women than in men, especially in women, who are older than 55 years (Brush et al., 2020[6]). Sex-specific differences in AMI patients exist not only in the presentation of acute symptoms but also in the pathophysiological mechanisms and especially in the outcomes (Mehta et al., 2016[16]). Thus within a year after the first AMI, more women than men will die (at ≥ 45 years of age: 23 % of women and 18 % of men). Also within a period of 5 years after the first AMI, more women than men will die (47 % of women and 36 % of men) or develop heart failure (at > 45 years of age: 22 % of women and 16 % of men) (Mozaffarian et al., 2016[18]).

### Trimethylamine N-oxide (TMAO)

One potential biomarker of cardiovascular risk, which interferes with many important physiological regulatory pathways, is trimethylamine-N-oxide (TMAO). This small organic molecule is generated by the gut microbiome, and alterations in the gut microbial community structure might also have an impact on TMAO production (Wu et al., 2017[26]). Intestinal bacteria metabolize L-carnitine- and phosphatidylcholine-containing nutrients, such as eggs, cheese and red meat, to trimethylamine (TMA), which is subsequently absorbed into the bloodstream and further oxidized to TMAO by the hepatic flavin-containing monooxygenase (FMO) family members FMO1 and FMO3 (Tang et al., 2013[22]; Bennett et al., 2013[5]). Since microbiome-dependent TMAO has become a predictor of various diseases (i.e., cardiovascular disease, chronic kidney disease, Alzheimer's disease), the quantitative determination of this biomarker is of high interest to clinicians (Wu et al., 2017[26]; Tang et al., 2013[22]; Zheng et al., 2019[27]; Wilson et al., 2015[25]).

### Trimethylamine N-oxide (TMAO), atherosclerosis and acute myocardial infarction (AMI)

TMAO has been shown to be an important protein stabilizer that preserves protein folding. This small molecule acts as an electron acceptor to enhance oxidative stress, subsequently leading to vascular inflammation (Ufnal et al., 2015[23]; Li et al., 2017[13]). Furthermore, this metabolite is known to promote endothelial cell senescence, platelet hyperreactivity and thrombosis (Zhu et al., 2016[28]). In addition, a recent study by Li et al. demonstrated that the blood concentration of TMAO is significantly associated with the grade of vascular calcification in AMI patients (Li et al., 2021[12]). Since TMAO is involved in these pathophysiological processes, high circulating blood levels of this biomarker may enhance the progression of atherosclerosis with the subsequent development of severe cardiovascular diseases, such as the acute coronary syndrome (Querio et al., 2019[19]). Moreover, individuals with low TMAO levels have been shown to have a lower risk of coronary artery disease (Liu et al., 2021[14]). A low TMAO concentration was associated with a better diet quality, characterized by a lower dietary intake of red meat and a higher dietary intake of whole grains and fibre (Liu et al., 2021[14]). 

Recently, microbiota-derived pro-atherogenic TMAO was also shown to be a useful parameter in the management of the post-procedural care of patients with AMI (Żurawska-Płaksej, et al., 2019[29]). Serum TMAO levels after AMI were associated with coronary plaque complexity and progression and significantly predicted future cardiovascular events in AMI patients (Matsuzawa et al., 2019[15]). Although all these studies address the involvement of TMAO in the pathophysiology of AMI, possible sex-specific aspects of this biomarker were not considered in the study designs.

### Objectives

AMI is more frequently misdiagnosed in women than in men. This might be explained by not only sex-specific differences in symptom manifestations but also sex-dependent differences in the pathophysiological processes underlying the development of coronary artery disease and AMI (Brush et al., 2020[6]). In addition, during the first years after AMI, women also have a significantly higher mortality rate than men (Mozaffarian et al., 2016[18]).

To obtain a deeper understanding of AMI and the associated morbidity and mortality, these sex-specific differences must be taken into account. TMAO plays a significant role in the pathophysiology and prognosis of coronary artery disease. Although TMAO might have less clinical significance in acute cardiac events, it is assumed that persistently high TMAO levels might significantly enhance the risk of severe cardiac events in the future (Alhmoud et al., 2019[1]). Previous study data in non-cardiac patients with carbohydrate malabsorption suggest sex differences in TMAO blood concentrations (Meinitzer et al., 2020[17]). Thus, the aim of this study in AMI patients was to determine for the first time whether there are also sex-specific differences in TMAO concentrations immediately after AMI as well as before and after cardiac rehabilitation.

## Materials and Methods

### Participants and procedures

All 56 study participants were drawn from AMI inpatients hospitalized in the Division of Cardiology, Department of Internal Medicine, Medical University of Graz, Austria. The overall sample consisted of 45/56 (80.4 %) males and 11/56 (19.6 %) females, and all the AMI patients were Caucasian. The mean age was 57.9 (± 12.0) years. The types of cardiac rehabilitation included outpatient and inpatient rehabilitation. The rehabilitation centres were specialized in cardiac rehabilitation, and the cardiac rehabilitation objectives included recommended lifestyle modifications, such as increased physical exercise combined with restricted dietary intake and dietary changes. The average waiting period prior to the start of rehabilitation after AMI was 5.5 (SD: + 4.4) weeks. The duration of cardiac rehabilitation was 4 weeks. 

For the assessment of TMAO, serum samples were collected within the first day after hospital admission due to AMI and at the start and end of cardiac rehabilitation.

The exclusion criteria for this study were additional acute and severe somatic illnesses other than AMI, delirium and pre-existing Alzheimer's disease.

### Ethics statement

This research project was approved by the Institutional Review Board of the Medical University of Graz (Approval number: 28-126 ex 15/16). Data protection measures met the standards set by Austrian law. All assessments were carried out in accordance with the approved guidelines, and all participants in this study had to provide signed informed consent. Subjects could decide to withdraw from this study project at any time.

### Socio-demographic characteristics and anthropometry

Regarding the socio-demographic characteristics, age and sex were recorded at the time of hospital admission due to AMI. The collected anthropometric data included height (cm) and weight (kg).

### Cardiac status at the time of inpatient admission due to AMI, percutaneous coronary intervention (PCI)-related parameters, in-hospital outcome, cardiac risk factors in AMI patients and renal function

The following clinical data were obtained to characterize the study sample:

- Cardiac status at the time of inpatient admission due to AMI: type of myocardial infarction (STEMI anterior; STEMI posterior; NSTEMI), Killip class (= post-AMI mortality risk stratification; Killip I: no clinical signs of heart failure, Killip II: rales or crackles in the lungs, S3 heartbeat, and elevated jugular venous pressure, Killip III: acute pulmonary edema, Killip IV: cardiogenic shock or hypotension/systolic blood pressure < 90 mmHg), abnormal level of troponin T, and AMI-related resuscitation.
- Percutaneous coronary intervention (PCI): coronary artery disease (number of affected vessels); thrombolysis in myocardial infarction flow (TIMI) before and after PCI, and multi-vessel PCI.
- In-hospital outcomes: death, major bleeding, reinfarction.
- Cardiac risk factors: nicotine abuse, peripheral arterial occlusive disease, insulin-dependent diabetes mellitus (IDDM), non-insulin-dependent diabetes mellitus (NIDDM), hypertension, hyperlipidemia, relevant family history, previous myocardial infarction.
- Renal function: The glomerular filtration rate (GFR), as an indicator of renal function, is well known to have an impact on TMAO levels.

### Laboratory analyses

TMAO was analyzed with the use of a stable isotope dilution assay and high-performance liquid chromatography (HPLC) with electrospray ionization tandem mass spectrometry on a SCIEX QTRAP 4500 triple quadrupole instrument (Applied Biosystems, Framingham, MA, USA) equipped with an Agilent 1260 Infinity HPLC system (Agilent Technologies, Santa Clara, CA, USA). The intra- and inter-day coefficients of variation (CVs) ranged between 2.2 and 3.4 % and 6.9 and 9.9 %, respectively (Enko et al., 2020[7][8]). The estimated GFR was calculated with the Chronic Kidney Disease Epidemiology Collaboration (CKD-EPI)-GFR equation (Levey et al., 2009[11]).

### Statistical analyses

Descriptive statistics were conducted based on socio-demographic, clinical and treatment-related data and are presented as means and standard deviations (SDs). TMAO concentrations in female and male AMI patients were analyzed with an analysis of covariance (ANCOVA), in which age, renal function (GFR), coronary artery disease (number of affected vessels) and type of cardiac rehabilitation (outpatient versus inpatient) were included as covariates.

All statistical analyses were performed with SPSS 25.0 for Windows (SPSS; Chicago, IL).

## Results

### Trimethylamine N-oxide (TMAO)

Shortly after hospital admission due to AMI, male patients initially had significantly lower TMAO levels than female patients. Prior to the start of cardiac rehabilitation, the TMAO levels were significantly elevated in males. However, during rehabilitation, this TMAO elevation did not increase further and remained stable. In contrast, female AMI patients had significantly higher TMAO levels within the first day after hospital admission due to AMI. These initially high TMAO levels remained almost unchanged in the female AMI patients until the start of cardiac rehabilitation and only reached the lower TMAO levels observed in the male patients after rehabilitation. [ANCOVA: TMAO (time, Greenhouse-Geisser) F = 3.379, df = 1.77, p = 0.044; TMAO (sex) F = 4.830, df = 1, p = 0.033; TMAO (sex x time, Greenhouse-Geisser) F = 4.625, df = 1.77, p = 0.015; covariate age x time (Greenhouse-Geisser): F = 2,850, df = 1.77, p = 0.070; covariate type of rehabilitation x time (Greenhouse-Geisser): F = 0.647, df = 1.77, p = 0.508; covariate renal function (GFR) x time (Greenhouse-Geisser): F = 3.862, df = 1.77, p = 0.029; covariate coronary artery disease (number of affected vessels) x time (Greenhouse-Geisser): F = 1.383, df = 1.77, p = 0.256].

Raw data are provided in Supplementary Table 1.

**Figure 1:**
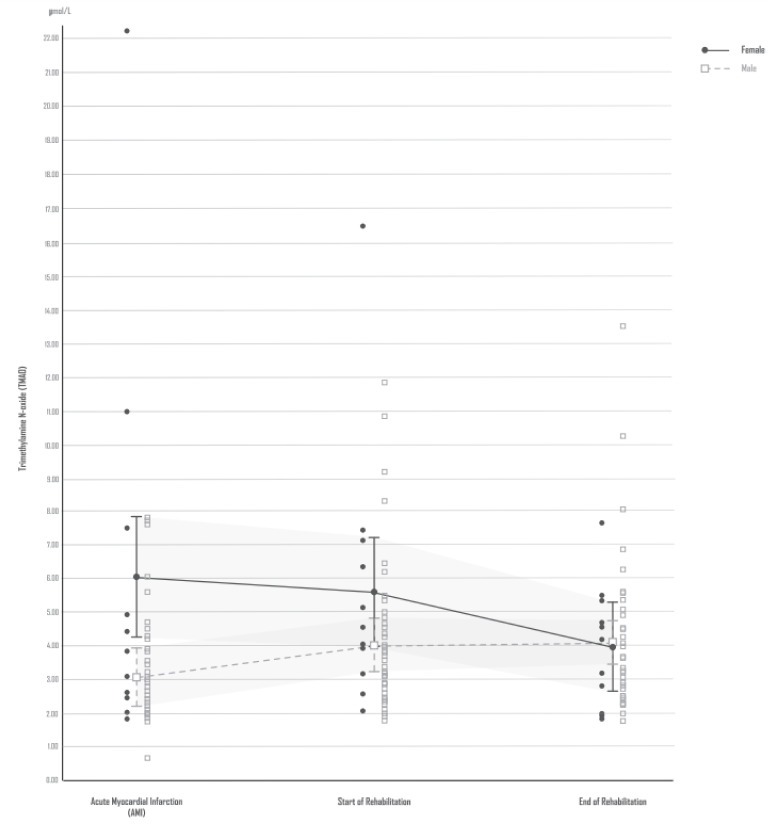
TMAO blood concentrations in female and male AMI patients

**Figure 1**(Fig. 1) shows the TMAO blood concentrations in female and male AMI patients shortly after hospital admission due to AMI and at the start and end of cardiac rehabilitation.

**Table 1**(Tab. 1) presents the socio-demographic characteristics, cardiac status at the time of AMI, PCI-related parameters, in-hospital outcome, cardiac risk factors, renal function and cardiac rehabilitation characteristics of female and male AMI patients.

## Discussion

### TMAO serum levels and adverse cardiovascular events

TMAO is predictive of adverse cardiovascular events, including myocardial infarction (Tang et al., 2013[22]; Baranyi et al., 2021[3]). A dose-response meta-analysis by Schiattarella et al. (2017[21]), based on seven studies, also showed that the relative risk (RR) for all-cause mortality significantly increases by 7.6 % per 10 μmol/L increment of TMAO [summary RR: 1.07, 95 % CI (1.04-1.11)] (Schiattarella et al., 2017[21]).

### Sex-specific differences in TMAO concentrations 

To the best of our knowledge, this is the first study addressing possible sex-specific differences in individuals after AMI. In comparison, two previously published studies that reported TMAO as a possible predictive parameter of biochemical and cardiovascular status after AMI unfortunately did not consider sex-specific influences on this biomarker (Żurawska-Płaksej et al., 2019[29]; Matsuzawa et al., 2019[15]).

In the current study, female patients were found to have significantly higher TMAO blood concentrations within the first day after AMI than male patients. This sex-specific difference persisted and remained significant until the beginning of cardiac rehabilitation and disappeared by the end of cardiac rehabilitation. Males and females were observed to have similar TMAO profiles after rehabilitation. 

Sex differences may play essential roles in the interpretation of TMAO blood concentrations. It is possible that women are at higher thrombotic risk than men because of their higher TMAO blood levels and the consequently higher level of TMAO-initiated activation of platelets (Zhu et al., 2016[28]; Razavi et al., 2019[20]). Moreover, higher hepatic FMO3 expression in females could be another possible mechanism underlying a faster TMAO production in women than in men (Razavi et al., 2019[20]). In mouse experiments, female animals produced much more TMAO than male animals (Veeravalli et al., 2018[24]). One explanation of this phenomenon is that FMO3, which is the major enzyme involved in the TMA to TMAO conversion in humans and mice, is switched off in the liver of male but not female mice at the age of 5-6 weeks (Veeravalli et al., 2018[24]; Falls et al., 1995[10]). It could be speculated that this limiting effect of FMO3 on the conversion of TMA to TMAO in males might also be one possible mechanism explaining the sex-specific TMAO levels in humans.

### TMAO levels, cardiac rehabilitation and lifestyle modifications

According to a study by Erickson et al. in 2019, physical exercise alone did not significantly reduce TMAO levels; however, caloric restriction combined with exercise promoted a reduction in TMAO (Erickson et al., 2019[9]). Erickson et al. (2019[9]) further suggested that a hypocaloric diet led to a reduction in the absolute levels of dietary precursors, such as TMA, which partially might explain the decrease in TMAO (Erickson et al., 2019[9]).

Differences in TMAO blood concentrations may also occur due to differences in dietary intake (lower dietary intake of red meat and a higher dietary intake of whole grains and fibre) (Razavi et al., 2019[20]; Erickson et al., 2019[9]). Thus, adherence to the Mediterranean diet is known to be associated with lower TMAO levels (Barrea et al., 2019[4]).

In this study, cardiac rehabilitation objectives included recommended lifestyle modifications such as increased physical exercise combined with restricted dietary intake and dietary changes. While female patients benefitted from the reduction in their initially high TMAO levels during cardiac rehabilitation, male AMI patients also benefitted during rehabilitation from the prevention of further TMAO increases after AMI. 

Therefore, regarding TMAO, the benefits of cardiac rehabilitation observed in both sexes are probably due to microbiome changes and cardiac rehabilitation associated lifestyle modifications, especially caloric restriction and additional dietary changes, such as reductions in the consumption of L-carnitine- and phosphatidylcholine-containing nutrients (e.g., eggs, cheese, and red meat).

### Limitations

Several limitations of this study must have to be mentioned. The patient population was relatively small, and the possible effects of food intake were not assessed. Precursors of TMAO (i.e., choline, betaine, L-carnitine, TMA) were not measured. The sex-specific differences found in this work should be investigated further in a larger study group and for a longer period of observation.

## Conclusions

After AMI, women might be at higher cardiovascular risk due to persistently higher levels of TMAO. However, both sexes might benefit from cardiac rehabilitation with lifestyle modifications, such as increased physical exercise combined with restricted dietary intake and dietary changes. Thus, high TMAO levels in women might decrease after cardiac rehabilitation due to rehabilitation-associated lifestyle modifications. These lifestyle modifications after AMI might also prevent increases in TMAO concentrations in men.

## Declaration

### Conflict of interest

Hanns Harpf, Leonhard Harpf and Heimo Traninger have a financial relationship with the organization that sponsored the research. All other authors declare that they have no disclosures.

### Funding

This work was supported by the ZARG, Zentrum für ambulante Rehabilitation GmbH, Graz, Austria under unrestricted research grant 4325/Medical University of Graz.

### Authorship confirmation statement

All authors are responsible for a significant part of the manuscript. All authors have taken part in writing the manuscript, reviewing it, and revising its intellectual and technical content. 

### Acknowledgments

All authors had full access to all the data in the study and take responsibility for the integrity of the data and the accuracy of the data analysis.

## Supplementary Material

Supplementary data

## Figures and Tables

**Table 1:**
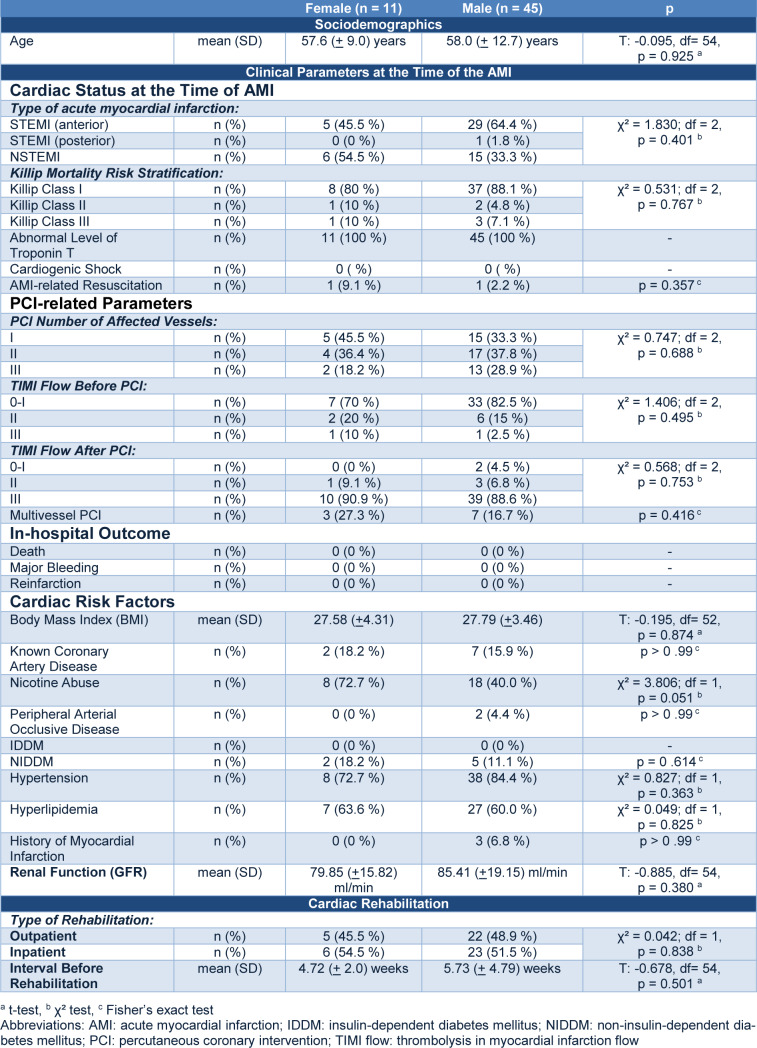
Socio-demographic characteristics, cardiac status at the time of AMI, PCI-related parameters, in-hospital outcome, cardiac risk factors, renal function and cardiac rehabilitation characteristics of female and male AMI patients.
